# Association of four differently processed diets with plasma and urine advanced glycation end products and serum soluble receptor for advanced glycation end products concentration in healthy dogs

**DOI:** 10.1111/jpn.13927

**Published:** 2024-01-27

**Authors:** Siobhan Bridglalsingh, Stephanie Archer-Hartmann, Parastoo Azadi, Claire Barbier de La Serre, Rebecca L. Remillard, Gregory D. Sunvold, Joseph W. Bartges

**Affiliations:** 1 Department of Small Animal Medicine and Surgery, University of Georgia, Athens, Georgia, USA; 2 Analytical Services, Complex Carbohydrate Research Center, University of Georgia, Athens, Georgia, USA; 3 Department of Biomedical Sciences, Colorado State University, Fort Collins, Colorado, USA; 4 Veterinary Nutritional Consultations, Inc., Hollister, North Carolina, USA; 5 Sunvold Technology, LLC, Lewisburg, Ohio, USA

**Keywords:** advanced glycation end products, dogs, Maillard reaction, pet food processing, soluble receptor for advanced glycation end products (sRAGE)

## Abstract

Advanced glycation end products (AGEs), formed via the Maillard reaction (MR) during processing of foods, have been implicated in inflammatory and degenerative diseases in human beings. Cellular damage is primarily caused by AGE binding with the receptor for AGEs (RAGE) on cell membranes. An isoform of RAGE, soluble RAGE (sRAGE), acts as a decoy receptor binding circulating AGEs preventing cellular activation. Pet food manufacturing involves processing methods similar to human food processing that may increase dietary AGEs (dAGEs). We hypothesized that diet, plasma and urine AGEs, and serum sRAGE concentrations would differ between thermally processed diets. This study examined the association of four differently processed diets: ultra-processed canned wet food (WF); ultra-processed dry food (DF); moderately processed air-dried food (ADF) and minimally processed mildly cooked food (MF) on total plasma levels of the AGEs, carboxymethyllysine (CML), carboxyethyllysine (CEL), methylglyoxal hydroimidazolone-1, glyoxal hydroimidazolone-1, argpyrimidine, urine CML, CEL and lysinoalanine, and serum sRAGE concentration. Ultra-high-performance liquid chromatography–tandem mass spectrometry was used to measure AGEs. sRAGE concentration was measured using a commercial canine-specific enzyme-linked immunosorbent assay kit. Total dAGEs (mg/100 kcal as fed) were higher in WF than in other diets. Plasma total AGEs (nM/50 μL) were significantly higher with WF, with no difference found between DF, ADF, and MF; however, ADF was significantly higher than MF. Urine CML (nmol AGEs/mmol creatinine) was significantly higher with DF than with WF and MF. There were no significant differences in total urine AGEs or serum sRAGE concentration between diets. In conclusion, different methods of processing pet foods are associated with varied quantities of AGEs influencing total plasma AGE concentration in healthy dogs. Serum sRAGE concentration did not vary across diets but differences in total AGE/sRAGE ratio were observed between MF and WF and, ADF and DF.

## 1 | INTRODUCTION

The Maillard reaction (MR) is a series of spontaneous, non-enzymatic reactions that occur in heat-treated foods between a carbonyl group of reducing sugar and the amino group of an amino acid to produce dark-pigmented melanoidins (ALjahdali & Carbonero, 2017). This ‘browning’ (Maillard, 1912) reaction gives cooked food its characteristic colour, aroma and flavour while increasing palatability and desirable appearance (Friedman, 1996). Thermal processing of foods increases food safety by reducing bacterial numbers and increasing storage times (Poulsen et al., 2013). Advanced glycation end products (AGEs), produced in the late stage of the MR consist of a large number of heterogeneous compounds that are formed between sugars and amino acids, proteins, lipids and nucleic acids via the MR (Uribarri et al., 2015; Zhu et al., 2018). The most commonly measured AGEs in foods, biofluids and tissues are N^ε^-carboxymethyllysine (CML), N^ε^-carboxyethyllysine (CEL), methylglyoxal hydroimidazolone-1 (MG-H1), pentosidine, glucosepane, and pyrraline (Kellow & Coughlan, 2015).

In human beings, increased consumption of AGEs is associated with the ‘Western diet’ and related lifestyle diseases such as obesity, type 2 diabetes mellitus, cardiovascular disease, atherosclerosis, systemic hypertension and stroke (Mirmiran et al., 2018; Nowotny et al., 2018; Šebeková & Brouder Šebeková, 2019). Foods included in the Western diet are thermally processed, relatively low cost, convenient to serve and readily available to the consumer (Delgado-Andrade, 2014). High AGE quantities exist in these commonly consumed food items suggesting a potential role in the pathogenesis of diseases (Chaudhuri et al., 2018).

The AGE pool in the body, composed of physiologically produced endogenous AGEs and exogenous dietary AGEs (dAGEs), increases the potential for inflammation and oxidative stress. These two sources are indistinguishable from each other in structure and function, and gradually accumulate in tissues (Uribarri et al., 2015). There are three broad categories of pathophysiological effects of high AGE tissue accumulation (Kellow & Coughlan, 2015): (1) protein crosslinking causing vascular dysfunction related to ageing and diabetes mellitus due to AGE modification of protein structure and function; (2) formation of reactive oxygen species (ROS) via AGE catalytic activity at sites of their accumulation; and (3) binding and activation of a range of receptors, especially the receptor for advanced glycation end products (RAGE), that initiates a cascade of inflammatory reactions and release of proinflammatory mediators, which sustain inflammation and oxidative stress.

The most significant mechanism by which AGE-induced inflammation is initiated and sustained is via AGE-RAGE binding (Ramasamy et al., 2012). RAGE is a member of the immunoglobulin G (IgG) superfamily (Gupta et al., 2018) and is a multi-ligand cell surface pattern recognition receptor (PRR) with toll-like receptor (TLR) activity (Kellow & Coughlan, 2015). Two functionally equivalent isoforms of RAGE, endogenous secretory RAGE (esRAGE) and cleaved RAGE (cRAGE), have been identified as decoy receptors that attenuate the effects of cellular AGE-RAGE interaction by binding to AGEs in circulation. Both isoforms are known as soluble RAGE (sRAGE) and the terms are often used interchangeably (Ciccocioppo et al., 2015; Maillard-Lefebvre et al., 2009; Zhang et al., 2008). Studies show altered concentrations of these forms of the receptor in chronic inflammatory conditions (Bierhaus et al., 2005) such as inflammatory bowel disease (Heilmann et al., 2014; Meijer et al., 2014), ulcerative colitis and Crohn’s Disease (Ciccocioppo et al., 2015) with concurrent increase of gastrointestinal RAGE expression in humans, mice (Body-Malapel et al., 2019), and dogs (Cabrera-Garcia et al., 2021).

Commercial pet food processing uses thermal processing with varying amounts of moisture to give pet food its characteristic shape, colour, texture and appearance (van Rooijen et al., 2013). The MR occurring in pet foods promotes the formation of AGEs reducing the bioavailability of amino acids such as lysine, that are utilized as reactants (van Rooijen et al., 2013).

To study the effects of pet diet processing there is a need to differentiate and define the degree of heat and processing of both pet food ingredients and the final product. In the human nutrition literature, studies of dietary effects use processing categorization systems such as the NOVA classification system that categorizes human food and ingredients into four groups according to the degree of processing (Monteiro et al., 2019). One author suggested applying the NOVA categories, ‘minimally processed’ (MP) and ‘ultra-processed’ (UP) to commercial pet foods. Ultra-processed pet foods include foods and ingredients undergoing several processing steps and high heat conditions such as retorting, extrusions or pelleting whereas minimally processed pet foods include foods and ingredients subjected to one to two processing steps involving mild cooking conditions. Such minimally processed commercial pet foods require storage by refrigeration or freezing to reduce bacterial proliferation and to keep the product stable for consumption (Raditic, 2021). We suggest adding a third category called ‘moderately processed’ pet foods or ingredients to include entire diets or specific ingredients that undergo intermediate thermal processing.

We hypothesized that ultra-processed, moderately processed and minimally processed pet foods would have different levels of AGEs and that diets high in AGEs would be associated with higher plasma and urine AGE concentrations in dogs while serum sRAGE concentration would have an inverse relationship with dAGE quantity.

## 2 | MATERIALS AND METHODS

### 2.1 | Animals

Eight (intact) purpose-bred laboratory colony Beagle dogs (Summit Ridge Farms, Susquehanna, PA), four males and four females, ranging from 3 to 7 years old were used. Before entry into study, the health status of these animals was confirmed through results of physical examination, complete blood cell counts, serum biochemical analyses and urinalyses. Clinical parameters recorded for each dog included weekly body weights for each diet and the % change determined at the end of the 4-week feeding. Daily food consumption for each diet was recorded and averages were tallied per week and over 4 weeks. Diet portions were fed according to initial body weight (70 × body weight(kg)^0.75^) and modified weekly to maintain body weights within 5% of baseline (Table 1). All dogs were reported to be at ideal body condition (body condition score 5/9) and were maintained throughout the study. Blood and urine samples were collected at the end of feeding each diet (Weeks 4, 8, 12 and 16) for haematology, serum chemistry and urinalysis. Dogs were housed individually in cages under a 12-h light-dark cycle with temperature range maintained between 50°F and 85°F. Cages and bowls were cleaned and sanitized daily. Fresh tap water fit for human consumption was available ad libitum for the duration of the study period. This study was approved by the Summit Ridge Farms Institutional Animal Care and Use Committee (approval date and number – 5/10/2017; Protocol Title: UGAUFCC 00117).

### 2.2 | Diets

Four thermally processed diets of similar nutrient composition consisting of kangaroo meat and sweet potato that were differently processed were evaluated: 1 – an ultra-processed, retorted wet food (WF); 2—an ultra-processed, extruded dry food (DF); 3—a moderately processed air-dried food (ADF); and 4—a minimally processed mildly cooked food (MF). A *Pediococcus acidilactici* fermentation product was added to both ADF and MF as a biological preservative with bactericidal activity. A comparison of ingredients (Table 2) and nutrient profiles for each diet is presented (Table 3). The nutrient profile for MF without the addition of water is presented in Supporting Information: Table S1. The MF diet required protein, carbohydrate and water to be mixed immediately before feeding in the proportion 56:8:36 (Table 3). The WF and DF were processed at temperatures greater than 200°F (93°C) while ADF and MF were processed at temperatures under 150°F (65.5°C) (Table 4). These diets met the requirements for dog foods appropriate for adult maintenance as established by the Association of American Feed Control Officials (AAFCO).

### 2.3 | Experimental design

A Latin-square design was used to minimize differences and compare diets consumed by the same dog. Four diet sequences were created for feeding to avoid differences due to the order of consumption of diets such that each dog ate each diet. Pairs of dogs were randomly assigned to one of four diet group sequences. Each pair received the first diet in their sequence for 4 weeks, samples were collected and then dogs were crossed over to the next diet, without a washout period, until all dogs completed each diet in the predetermined sequence. Samples were collected before beginning the sequences of feeding to obtain a record of baseline data. The WF and MF diets were offered twice daily for a minimum of 1 h. The DF and ADF diets were offered once daily for a minimum of 1 h.

### 2.4 | Sample collection

#### 2.4.1 | Food samples

A single batch of food of each type was manufactured for the entire study period and stored at −80°C until feeding.A sample of the diets was collected at the beginning of each 4-week feeding period, shipped overnight on dry ice then stored at −80°C until analysis. For analysis of protein-bound AGEs in food samples, composite samples were made for each of the diets for a total of four samples per diet.

#### 2.4.2 | Plasma, serum and urine samples

Blood was collected before feeding (baseline) and once weekly at the end of the 7-day period before the morning feeding via jugular venipuncture in sterile syringes. Samples were divided into red-top serum separator tubes and green-top heparinized tubes (3 mL each). Each sample was centrifuged at 4°C for 15 min at 3000 RPM after clotting (red) and mixing (green) so that serum and plasma could be obtained via pipette and stored in cryovials at −70°C.

Urine was collected at the start of feeding (baseline) and once weekly at the end of the 7-day period before the morning feeding. Samples were obtained by placing the dogs in metabolism cages with open floors over stainless steel pans connected to glass jars. Urine was collected via cystocentesis if the urinary bladder could be palpated the following morning before feeding or from the jars that contained urine voided overnight. Urine samples were obtained using sterile syringes (4–6 mL), placed in cryovials for AGE analysis and stored at −70°C.

All samples for AGE analyses were shipped on dry ice and stored at −80°C at the laboratory until analysis. Free AGEs in plasma and urine were measured using ultra-high-performance liquid-chromatography mass spectrometry (UPLC-MS) and serum samples were used for quantitative analysis of canine sRAGE using a commercial enzyme-linked immunosorbent assay (ELISA) kit. Urine creatinine was measured using the modified Jaffé reaction in the Veterinary Clinical Pathology Laboratory at the University of Georgia.

### 2.5 | Analysis of AGEs in food and plasma

#### 2.5.1 | Food samples—CML, CEL and MG-H1 assay

##### Materials

Boric acid, sodium borohydride, trifluoroacetic acid (TFA), 1-butanol, methanol, sodium hydroxide, hydrochloric acid (HCl), water and acetonitrile were obtained from Sigma Aldrich. N^ε^-(1-carboxymethyl)-L-lysine (CML) and N^ε^-(1-carboxyethyl)-L-lysine (CEL) were obtained from Cayman Chemicals. All other reagents and solvents were of analytical or UPLC-MS grade.

##### Sample preparation

The diet sample preparation method was adapted and modified from work done on human foods investigating protein-bound AGEs (Scheijen et al., 2016). Approximately 6 g of each food sample was soaked in 40 mL of water for 72 h at 4°C to aid in homogenization. These samples were then homogenized using a commercial food processor and lyophilized to dryness. For analysis, 50 mg of each dried food sample for each of the 4-week feeding periods was used. All diets were <20% fat eliminating the need for a defatting step. All samples were mixed and deproteinized with 1000 μL cold (4°C) TFA, centrifuged (4300*g*, 4°C, 20 min) and the supernatant removed with a Pasteur pipette. Subsequent hydrolysis was performed by addition of 1000 μL 6 N HCl and incubation at 110°C for 24 h. After hydrolysis, 80 μL of hydrolysate was evaporated to dryness under nitrogen gas at 70°C. After drying, samples were derivatized with 100 μL 1-butanol:HCl (3:1 v/v) for 90 min at 70°C. Samples were then evaporated to dryness under nitrogen, redissolved in 300 μL water, vortexed and centrifuged at 15,000*g* for 20 min. For UPLC-MS analysis, 100 μL of sample was transferred to each of the two sample vials for analysis as duplicates.

##### Instrumentation

Liquid chromatography–tandem mass spectrometry (LC-MS/MS) was performed on an Orbitrap Elite Mass Spectrometer (Thermo Fisher Scientific) coupled to an RSLC liquid chromatography system (Thermo Fisher Scientific), and equipped with an electrospray ion source. Prepared samples were injected (volume 20 μL) onto the separation column (Waters Acquity UPLC BEH C18—2.1×150 mm^2^ with 1.7 μm particle size). The separation was performed under a linear gradient from 5% to 100% B (solvent A—100% water with 0.1% formic acid; solvent B—80% acetonitrile with 0.1% formic acid) at a flow rate of 0.3 μL/min. Electrospray was carried out in positive ion mode, and programs monitoring the expected ion masses (SIM) as well as fragmentation of those masses (SRM – CID) were carried out using the Thermo Fisher Excalibur software. The chromatograms for the CML and CEL internal standards were used to construct calibration curves, ranging from 1 to 100 μM concentrations of CML and CEL to calculate the quantity of all three AGEs in the diets: absolute CML and CEL concentrations and relative concentration of MG-H1 to the CML internal standard, and to confirm identification by retention time. Four replicates of each diet were analyzed for the AGEs: CML, CEL and MG-H1 (mg/100 kcal AF [as-fed]) corresponding to each 4-week feeding period of the crossover design.

#### 2.5.2 | Plasma samples—CML, CEL, MG-H1, glyoxal hydroimidazolone-1 and argpyrimidine assay

##### Materials

The internal standard (IS) N^ε^-(1-carboxymethyl)-L-lysine-d3 (CML-d3) was purchased from Cayman Chemical. A solution of picrylsulfonic acid (5% w/v) was purchased from Sigma Aldrich. Distilled water, methanol (MeOH) and formic acid (FA) were LC-MS grade. All other chemicals were of laboratory analytical reagent grade.

##### Sample preparation

The method was adapted from prior determination of free AGEs in rat plasma (Hashimoto et al., 2013) and, to the authors’ knowledge, is the first time it was applied to determine free AGE concentration in canine plasma. One modification was made following the derivatization step where 100 μL of 0.2% FA was added to the solution to stop the reaction, and 100 μL was placed into sample vials for injection.

##### Instrumentation

LC-MS/MS was performed on an Orbitrap QExactive Mass Spectrometer (Thermo Fisher Scientific) coupled to a Vanquish UPLC (Thermo Fisher Scientific), and equipped with an electrospray ion source. Prepared samples were injected (volume 20 μL) into the separation column (Agilent Zorbax Eclipse XDB-18—2.1 × 150 mm^2^, 1.8 μm). The separation was performed under a linear gradient from 0% to 100% B (solvent A—100% water with 0.1% formic acid; solvent B—100% MeOH with 0.1% formic acid) at a flow rate of 200 μL/min. Electrospray was carried out in positive ion mode and programs monitoring the expected ion masses (SIM) as well as fragmentation of those masses (PRM – HCD) were carried out using the Thermo Fisher Excalibur software. Concentration of AGEs in the sample vials was calculated as a peak area ratio of the unlabelled peak to the CML internal standard peak area. Measurement of plasma AGEs was done using samples in duplicate and reported as absolute quantities of CML, for which an IS was used, in addition to CEL, glyoxal hydroimidazolone-1 (GH-1) and MG-H1 and argpyrimidine (AP) all relative to the concentration of CML in samples. The limit of detection was >0.16 nM and the limit of quantification (LOQ) was >0.47 nM for all five plasma AGEs for this assay (Hashimoto et al., 2013).

#### 2.5.3 | Urine samples—CML, CEL and LAL assay

##### Materials

The IS used was N^ε^-(1-carboxymethyl)-L-lysine-d3 (CML-d3) and was purchased from Cayman Chemical. Distilled water, acetonitrile (ACN) and formic acid (FA) were LC-MS grade or higher. All other chemicals were of laboratory analytical reagent grade.

##### Sample preparation

Samples were prepared with minimal modifications from Palaseweenun et al. (2020). Briefly, an aliquot of 0.5 mL urine was thawed and filtered through a centrifugal filter (10 kDa MWCO; Pall). The sample was then diluted 50× into a solution containing 0.1% formic acid that contained 10^−3^ g/L CML-d3. All solutions were stored at −80°C for storage.

##### Instrumentation

LC-MS/MS was performed on an Orbitrap QExactive Mass Spectrometer (Thermo Fisher Scientific) coupled to a Vanquish UPLC (Thermo Fisher Scientific), and equipped with an electrospray ion source. Prepared samples were injected (volume 1 μL) into the separation column (Millipore SeQuant^®^ ZIC-HILIC 3.5 μm, 150 × 2.1 mm^2^). The separation was performed in a step-wide gradient where solvent A—100% water with 0.1% formic acid; solvent B—100% acetonitrile with 0.1% formic acid at a flow rate of 350 μL/min. The elution profile was as follows: 0–2 min isocratic with 90% B, 2–3 min linear gradient from 90% B to 65% B, 3–5 min isocratic on 65% B, 5–7 min linear gradient from 65% B to 40% B, 7–10 min isocratic on 40% B, 10–12 min linear gradient from 40% B to 90% B and 12–28 min isocratic on 90% B. Electrospray was carried out in positive ion mode and programs monitoring the expected ion masses (SIM) were carried out using the Thermo Fisher Excalibur software. The concentration of the AGEs was calculated as a ratio of the extracted masses of the CML-d3 peak to the biological AGE signals. The AGEs measured in the urine as duplicates were absolute quantities of CML for which an IS was used, and CEL and LAL, relative to the concentration of CML in the samples. Urine AGEs were expressed as a ratio of AGE concentration (nmol/L) to urine creatinine concentration (mmol/L) to account for variability in urine concentration.

### 2.6 | Analysis of canine RAGE in serum

Serum samples were used to measure sRAGE using the Commercial RayBiotech Inc.^®^ Canine RAGE ELISA kit. Samples were taken at the end of feeding each diet were analyzed according to the manufacturer’s instructions and plates were read using a BioTek Synergy^™^ HT microplate reader with Gen5^™^ Microplate Reader and Imager software. All serum samples were analyzed as duplicates. Standard curves were constructed and used to calculate sRAGE concentration (pg/mL) based on optical density at 450 nm.

### 2.7 | Statistical analyses

Data were analyzed using Analyse-It (v5.11.1) and IBM SPSS^®^ (Statistical Package for the Social Sciences, version 23) software platforms. The Shapiro–Wilk test revealed that data except clinical parameters were not normally distributed. AGE concentration in the diets (at the start of feeding—Weeks 1, 5, 9 and 13) was expressed as means and standard deviations. All clinical parameters were tested for statistical differences using a one-way analysis of variance for repeated measures with post hoc Bonferroni adjustment. Global differences in plasma and urine AGEs, sRAGE concentrations and, total plasma AGEs/sRAGE ratios across all diets were determined using non-parametric Friedman’s test for repeated measures. Statistically significant differences were determined using Wilcoxon–Mann–Whitney/Wilcoxon signed-rank test for individual comparisons. Differences were considered statistically significant at *p* < 0.05.

## 3 | RESULTS

### 3.1 | General characteristics of the laboratory colony study population

Serum chemistry and urinalysis results were within normal limits throughout the study. Statistical analyses revealed significant differences for several parameters between diets (Supporting Information: - Table S1) but all values were within normal reference intervals.

### 3.2 | Diet AGEs

There was no statistically significant difference in effect of time (χ^2^(3) = 5.01, *p* = 0.1713) on AGE concentration in diet samples over the 16-week study period. Thus, storage conditions did not significantly alter AGE quantity in diets and sequence of feeding the four diets did not influence results.

WF contained the highest amount of CML followed by ADF > DF > MF. In decreasing order, CEL was highest in WF > MF > DF > ADF. Relative quantities were obtained for MG-H1 based on CML concentration (IS). The highest mean amount of MG-H1 was seen with MF > DF > WF > ADF (Table 5).

Total AGEs can be described as the sum of the absolute quantities of CML and CEL as well as the sum of all three measured in the food recalling that in this study, MG-H1 concentration was calculated relative to CML concentration. The sum of CML and CEL matched quantities as for CML alone: WF > ADF > DF > MF. Total AGEs (sum of all 3) produced similar results described by mean values: WF > ADF > DF > MF (Table 5).

### 3.3 | Plasma AGEs

Plasma CML (Table 6a) was highest when ADF was consumed with lower amounts for DF and WF while MF was lowest. Plasma CML (Table 7a) revealed significant differences between MF and the other diets (Table 7b). Plasma CEL (Table 6a) showed WF associated with the highest plasma concentration followed in decreasing order by DF, MF and ADF. Significant differences (Table 7a) were found between WF and the other diets. Plasma dicarbonyl GH-1 was highest (Table 6a) with ADF, lower with WF and DF and lowest with MF. There were significant differences in plasma GH-1 (Table 7a) between ADF and MF in addition to WF and MF. Plasma MG-H1 (Table 6a) was higher than GH-1 found as highest with WF, lower with DF and ADF and lowest with MF. Plasma MG-H1 (Table 7a) showed significant differences between WF and other diets as well as between ADF and MF. Plasma AP (Table 6a) was highest with MF followed in decreasing order by WF, DF and ADF. Plasma AP was significantly different between MF and other diets (Table 7a).

Pairs, grouped and plasma AGE groups were compared between diets. For this study, CML and CEL (lysine adducts) and GH-1 and MG-H1 (dicarbonyls derivatives) were designated pairs, grouped designated as CML + CEL + GH-1+ MG-H1 and finally, total as CML + CEL + GH-1+ MG-H1 + AP. (Table 6b). Total CML + CEL was highest with WF (Table 6b) with significant differences between MF (lowest CML + CEL) and the other diets (Table 7b). For GH-1+ MG-H1, the high MG-H1 with WF influenced the results of the sum such that the amount was highest with WF (Table 6b). This influence of the relatively high amounts of MG-H1 in this pair resulted in significant differences between diets similar to MG-H1 alone. Plasma GH-1+ MG-H1 for WF was significantly different from other diets and ADF diet was significantly different from MF (Table 7b). When the plasma AGE group, CML + CEL + GH-1+ MG-H1, was compared with the total AGE group results were similar (Table 6b). The highest sum of the plasma lysine adducts and dicarbonyls alone and total plasma AGEs were observed with WF (Table 6b). Significant differences between diets were similar (Table 6b) when comparing the group of four AGEs with the total of all five AGEs. Significant differences were observed for plasma concentration of total AGEs with WF and other diets as well as between ADF and MF (Table 7b).

### 3.4 | Urinary AGEs

In decreasing order (Table 8) CML was highest with DF followed by ADF > WF > MF. This was different from the quantity of urine CEL where it was highest with WF and lower with DF > MF > ADF. WF was associated with the highest urinary excretion of LAL but unlike CEL, this was followed in decreasing order by MF > DF > ADF. When examining the sum of AGEs, totals were divided into CML + CEL as a sum of the lysine adducts, also measured in food and plasma, and as CML + CEL + LAL. When CML + CEL are considered, urinary excretion was highest with DF and lower in WF > ADF > MF. The sum of all three urinary AGEs revealed that the highest excretion was observed with DF followed by WF > ADF > MF. Friedman’s test revealed significant differences for CML only between DF and WF and DF and MF as seen in Table 9. There were no statistically significant differences in CEL, LAL or sum of these AGEs in the urine (Table 9).

### 3.5 | Serum sRAGE concentration

Friedman’s test revealed no statistically significant differences in serum sRAGE concentration between diets, χ^2^(3) = 7.35 (*p* = 0.062) although the highest quantity was found with MF (Table 10a). The total AGE/sRAGE ratios were determined for each diet (Table 10b) showing significant differences between MF and WF and between ADF and DF.

## 4 | DISCUSSION

### 4.1 | Clinical parameters of the canine colony

Dogs were clinically normal as confirmed by physical examination, and laboratory data at the start of and during the study. Statistical analysis revealed significant differences in a few parameters between diets; however, there is no clinical relevance of these differences since all were within normal reference intervals.

### 4.2 | Dietary AGEs

Results of our study support the hypotheses that processing, as one of several factors, may influence dAGE concentration and that dAGEs are associated with alterations in plasma and urine AGE concentrations. We evaluated the association of diet with plasma and urine AGEs and serum sRAGE using kangaroo-based, low-fat diets that were processed differently including commercially available DF and WF diets with ADF and MF diets. Diets differed primarily by method of production; however, while diets had some common ingredients and similar nutrient composition, they were not exactly alike due to the use of two commercially processed diets (WF and DF) and difficulties in producing identical ADF and MF diets (Niamnuy & Devahastin, 2010).

The first studies to determine AGE quantities in commercial pet food provided evidence not only of the extent of the MR in high-heat processed dog foods (van Rooijen et al., 2014a) but also documented decreased lysine bioavailability due to its consumption through glycation reactions (van Rooijen et al., 2014b). Fructoselysine (FL), CML and hydroxymethylfurfural (HMF), in canned food, were found to be higher on a mg/kg of diet on a dry matter basis when compared to pelleted foods. In one study, average daily dietary intake of HMF (mg/kg body weight^0.75^) was estimated to be 122 times higher for dogs consuming extruded commercial dog foods than for adult humans consuming a Western diet on a metabolic body weight basis (van Rooijen et al., 2014a). When considering dAGE contribution to the body’s AGE pool, it is the total AGE burden of the diet that may be of significance (Liang et al., 2020; Nowotny et al., 2018; Šebeková & Somoza, 2007).

In agreement with the work done by van Rooijen et al. (van Rooijen et al., 2014a), WF contained the highest total dAGEs. Although DF is also manufactured using high temperatures, it is lower in water content than WF. AGE formation during cooking is influenced by water content (Sherwin & Labuza, 2003), and low and high moisture decrease the rate of AGE formation by reducing the motion of molecules in a low moisture environment or by diluting reactants in a high moisture environment (Poulsen et al., 2013). The ADF, subjected to a lower temperature during drying, contained the second highest total AGEs for both the pair of lysine adducts and all three AGEs (Table 5) that could be attributed to the addition of dextrose to this diet as a substrate for *P. acidilactici* fermentation. It is possible that processing conditions did not favour fermentation of all added dextrose leaving this reducing sugar available for glycation reactions during processing resulting in increased total AGEs higher than that of DF. Further investigation into the effect of addition of bacterial fermentation products to moderately or minimally processed diets related to AGE formation is needed to determine the cause of the higher AGE content of ADF.

Comparison of total AGEs in these diets matches the findings of other studies that examined the AGE content of foods (Scheijen et al., 2016; van Rooijen et al., 2014a); however, information regarding pet foods is sparse. Our results are consistent with data derived from analysis of human foods where the lowest mean AGE concentration corresponded to mildly cooked food compared with the highest mean AGE concentration was associated with the food that was produced at high temperature for the longest period of time.

### 4.3 | Plasma and urine AGEss

Plasma CML and CEL concentrations were highest when dogs consumed WF, which contained the highest amounts of these AGEs. In addition to CML and CEL, we measured plasma glyoxal (GO) and methylglyoxal (MGO), which are alpha-dicarbonyls produced in the intermediate stage of the MR and are precursors of GH-1 and MG-H1. We measured downstream products of MGO including CEL, MG-H1 and AP (Wilker et al., 2001). While specific quantification of MGO is difficult because it is an unstable compound, formation of CEL + MG-H1 + AP is an estimate of its quantity in food samples and in plasma where both exogenous (dietary) and endogenous (physiological) forms contribute to the calculated concentration (Chakraborty et al., 2014). Plasma AP concentration was relatively low in plasma compared to the other MGO products with significant differences between MF and other diets. It is unknown why AP concentration would be highest when dogs were fed MF, but since this AGE was not assessed in the diets, its measurement could be considered in future experiments to determine if this is a consistent finding in mildly cooked diets and if there is any clinical significance of dietary intake in dogs. The sum of CML and CEL for all diets except WF exceeded the sum of GH-1 and MG-H1. Whether this is of clinical significance is unknown but given that dicarbonyl stress in the body can lead to the development of diseases in humans (Chaudhuri et al., 2018; Hellwig et al., 2018), and that there was a substantial amount of dicarbonyl derivatives in plasma associated with WF, it may be worthwhile to further investigate the existence of dicarbonyl stress in dogs on differently processed diets.

In human (Scheijen et al., 2018), dog (Palaseweenun et al., 2020) and cat (Palaseweenun et al., 2020; van Rooijen et al., 2016) studies, urinary excretion of free AGEs was an indicator of dAGE intake. Urine AGE concentration was expressed as a ratio with urine creatinine concentration to reduce variability due to glomerular filtration rate (GFR) and urine concentration at time of sample collection. Urine concentration of AGEs was higher when dogs consumed the lower moisture diets, DF and ADF, and was lower when dogs consumed the higher moisture diets, WF and MDF. Even though there were only differences noted for CML when DF was fed, this may indicate an effect of dry kibble diets on urine AGE excretion. One study suggests that there is variability in urine AGE excretion (Thornalley et al., 2003) that is dependent on the health status of the subjects and renal function. Healthy individuals may not have significant differences in urine AGE concentration but individuals with chronic disease may have higher urine AGE to urine creatinine ratios (del Carmen Hurtado-Sánchez et al., 2012). Future investigation into the effect of time, differences between spot versus pooled urine sample collection and urinary excretion of AGEs after consuming differently processed diets is recommended to better explore the influence of different diets on urine AGE concentration in dogs.

### 4.4 | sRAGE in serum

The results of the study did not support the hypothesis that higher dAGE intake and plasma concentration would be associated with lower sRAGE. In comparison, dAGE intake and serum sRAGE concentration also did not correlate in healthy human subjects (Van Puyvelde et al., 2014). Soluble RAGE concentration is highly variable in human subjects (Erusalimsky, 2021) and this was noted in this canine colony. Focus is now being placed on the evaluation of the AGE/sRAGE ratio as a risk factor for diseases (Prasad, 2019). While there were no statistical differences in absolute sRAGE concentration when dogs were fed the different diets, there were differences in AGE/sRAGE ratios between MF and WF and between ADF and DF. These statistical differences in combination with the lower sRAGE concentration corresponded to the consumption of WF and DF, which may suggest alterations in RAGE and sRAGE expression. It is possible that increased plasma AGE concentration was associated with increased sRAGE binding and clearance from the serum compared with ADF and MF as an early protective mechanism. Conversely, WF and DF may not stimulate increased sRAGE expression. Furthermore, dogs in this study were clinically healthy and the diets were fed for only 4 weeks. Evaluation of the AGE/sRAGE ratios as dogs consume long-term diets is needed. Changes in RAGE activity and serum sRAGE concentration may depend on persistently high AGE intake and accumulation within the body with concurrent predisposition to chronic diseases such as diabetes mellitus and renal disease or degenerative states. Prospective studies of canine subjects eating high AGE diets for their lifetime would allow observation of changes in AGE and sRAGE concentration or ratio that can be correlated with ageing and development of chronic disease.

There were several limitations to this study. Diets vary by processing method in addition to differences in ingredient and nutrient composition; therefore, differences in results cannot be solely attributed to the processing method. Future studies will focus on feeding identical diets that have been differently processed. The feeding regimen of the diets differed such that DF and ADF were fed once daily and WF and MF were fed twice daily. This resulted in samples being collected at different times after feeding, either 12 h or 24 h, which may have influenced plasma and urine AGE concentrations. Urine AGEs were determined in samples voided overnight or collected by cystocentesis before the morning meal, which may have altered urine composition due to the evaporation of voided samples. Collecting 24-h urine samples should be considered. Different AGEs were measured in food, plasma, and urine, thereby making it difficult to evaluate the influence of dAGEs on plasma and urine AGEs. Future studies should standardize feeding and sampling protocols, and measure the same AGEs in food, plasma, and urine.

## 5 | CONCLUSION

In conclusion, AGEs in dog foods, canine plasma and urine were identified and quantified using UPLC-MS. Total dAGEs in the four thermally processed pet foods were associated with alterations in the total plasma AGE concentration. The canned diet (WF) contained the highest amount of dAGEs that resulted in the highest total plasma AGE concentration with differences in the total plasma AGE/sRAGE ratios between WF versus MF and ADF versus DF. Differences in urine concentration of CML when DF was fed and differences in total plasma AGEs/sRAGE ratios between WF and MF and between ADF and DF were found. Further long-term feeding trials with measurement of AGEs in plasma, urine, intestinal mucosa and faeces are recommended to investigate the role and effects of AGEs in companion animals as part of comparative research.

## Supplementary Material

Supp for healthy dogs

## Figures and Tables

**TABLE 1:** Initial body weights of each dog at the start of feeding (baseline weight) each of the four diets.

Dog ID	Sex	WF	DF	ADF	MF
1	F	8.31	8.59	8.16	8.44
2	F	9.14	10.11	8.38	9.78
3	M	10.09	10.18	9.80	10.39
4	M	9.53	9.38	9.51	10.30
5	F	8.85	8.76	9.16	8.76
6	M	12.82	13.70	14.53	13.59
7	M	14.68	13.33	14.35	12.50
8	F	10.09	9.90	9.77	9.90
Mean		10.44	10.49	10.46	10.46
SD		2.187	1.956	2.530	1.762

Abbreviations: ADF, moderately processed air-dried food; DF, ultra-processed dry food; MF, minimally processed mildly cooked food; SD, standard deviation; WF, ultra-processed wet food.

**TABLE 2:** Formula per cent of ingredients of the four differently processed diets: ultra-processed wet food (WF), ultra-processed dry food (DF), moderately processed air-dried food (ADF) and minimally processed, mildly cooked food (MF).

Ingredient	Formula per cent			
	WF	DF	ADF	MF
Water	37.67	(as part of slurry)	8.53	8.53
Kangaroo meat	30.00		59.23	59.23
Sweet potato	14.00		26.84	26.84
Potato starch	6.85			
Kangaroo hearts	5.20			
Kangaroo liver	5.00			
Guar gum	0.30			
Vitamin premix	0.26		0.38	0.38
Sunflower oil	0.22	2.23	0.32	0.32
Tricalcium phosphate	0.15		0.22	0.22
Fructooligosaccharide	0.15	0.25	0.22	0.22
Salt	0.10		0.15	0.15
Potassium chloride	0.10		0.15	0.15
Dried potato		18.50		
Kangaroo slurry		18.50		
Dried chickpeas		18.50		
Pea protein		11.60		
Potato protein		11.60		
Green peas		9.72		
Sweet potato flour		3.00		
Liquid digest (flavour)		2.50		
Coconut oil		1.42		
Calcium carbonate		0.95	0.25	0.25
Dicalcium phosphate		0.55		
Salt		0.30		
Vitamin premix		0.12		
Choline chloride		0.10		
Mineral premix		0.06		
Natural preservative		0.05		
Taurine		0.04		
Mineral premix		0.02		
Dextrose			3.66	3.66
Bacterial cultures			0.05	0.05

**TABLE 3:** Comparison of the nutrient profiles of the four diets expressed on as fed, 100% dry matter (DM) and gram (g)/1000 kcal basis.

Nutrient	Units	WF			DF			ADF			MF		
		As fed	100% DM	g/1000 kcal	As fed (92% DM)	100% DM	g/1000 kcal	As fed (90% DM)	100% DM	g/1000 kcal	As Fed with water	100% DM	g/1000 kcal
ME (AAFCO – 3.5, 8.5 kcal/kg)		754.19	3717.29		3337.83	3628.08		3372.14	3746.83		1086.19	3367.19	
Moisture	%	79.71	0.00	1056.91	8.00	0.00	23.97	10.00	0.00	29.65	70.97	10.00	653.38
Protein	%	8.01	39.48	106.20	31.22	33.94	93.54	41.19	45.77	122.15	13.26	41.09	122.03
Fat	%	1.75	8.61	23.17	7.73	8.40	23.15	9.87	10.97	29.27	3.18	9.86	29.30
Ash	%	1.18	5.82	15.67	5.60	6.09	16.77	7.45	8.27	22.08	2.44	7.58	22.51
Crude fibre	%	0.22	1.11	2.97	2.07	2.25	6.21	1.49	1.65	4.40	0.48	1.48	4.40
AcDF	%	N/A	N/A	N/A	3.08	3.35	9.23	N/A	N/A	N/A	N/A	N/A	N/A
NDF	%	N/A	N/A	N/A	5.72	6.22	17.15	N/A	N/A	N/A	N/A	N/A	N/A
Carbohydrates	%	9.30	45.82	123.25	45.38	49.32	135.95	31.18	34.65	92.48	10.05	31.16	92.53
LA 18:2 n-6	%	0.24	1.17	3.14	3.11	3.38	9.33	1.17	1.30	3.46	0.38	1.18	3.50
ALA 18:3 n-3	%	0.03	0.13	0.36	0.15	0.16	0.44	0.15	0.16	0.44	0.05	0.15	0.44
AA 20:4 n-6	%	0.03	0.15	0.42	0.01	0.01	0.02	0.11	0.12	0.31	0.03	0.10	0.31
EPA 20:5 n-3	%	0.01	0.04	0.10	N/A	N/A	N/A	0.03	0.04	0.09	0.01	0.03	0.09
DHA 22:6 n-3	%	0.00	0.01	0.01	N/A	N/A	N/A	N/A	N/A	N/A	N/A	N/A	N/A
Total LCPUFA	%	0.02	0.09	0.23	N/A	N/A	N/A	0.07	0.08	0.22	0.02	0.07	0.22
Total n-6	%	0.27	1.33	3.59	3.12	3.39	9.34	1.28	1.43	3.81	0.42	1.29	3.84
Total n-3	%	0.06	0.31	0.83	0.15	0.16	0.45	0.33	0.36	0.97	0.11	0.33	0.97
Calcium	%	0.17	0.86	2.32	0.72	0.79	2.17	1.43	1.59	4.23	0.48	1.49	4.43
Phosphorus	%	0.16	0.76	2.06	0.60	0.66	1.81	0.83	0.92	2.47	0.27	0.83	2.47
Available phosphorus	%	0.15	0.74	1.99	N/A	N/A	N/A	0.80	0.89	2.38	0.26	0.80	2.38
Magnesium	%	0.02	0.08	0.22	0.14	0.16	0.43	0.10	0.11	0.28	0.03	0.10	0.29
Potassium	%	0.21	1.04	2.79	1.28	1.39	3.84	1.17	1.30	3.46	0.37	1.16	3.45
Sodium	%	0.10	0.48	1.28	0.18	0.20	0.55	0.51	0.57	1.52	0.16	0.51	1.51
Chloride	%	0.18	0.87	2.35	0.35	0.38	1.05	0.94	1.04	2.78	0.30	0.93	2.75
Iron	mg/kg	47.14	232.34	0.0625	246.39	267.81	0.074	206.00	228.89	0.06	66.88	207.33	0.06
Zinc	mg/kg	39.90	196.66	0.0529	197.88	215.09	0.059	214.73	238.59	0.06	69.51	215.49	0.06
Manganese	mg/kg	2.56	12.60	0.0034	65.42	71.11	0.020	12.78	14.20	0.00	4.16	12.90	0.00
Copper	mg/kg	2.57	12.66	0.0034	21.44	23.30	0.006	12.01	13.35	0.00	3.89	12.07	0.00
Iodine	mg/kg	0.42	2.05	0.0006	1.17	1.27	0.000	2.16	2.40	0.00	0.70	2.18	0.00
Selenium	mg/kg	0.17	0.82	0.0002	0.58	0.63	0.000	0.81	0.91	0.00	0.26	0.82	0.00
Vitamin A	IU/kg	8815.70	43,451.13	11,690.00	29,045.96	31,571.70	8700	21,570.74	23,967.51	6400.00	7017.84	21,755.30	6460.00
Vitamin D	IU/kg	182.00	897.05	240.00	2135.54	2321.24	640	943.72	1048.58	280.00	307.03	951.79	280.00
Vitamin E	IU/kg	69.06	340.37	90.00	454.87	494.43	140	360.98	401.09	110.00	117.38	363.88	110.00
Vitamin K	mg/kg	0.0025	0.01	0.0000	N/A	N/A	N/A	0.02	0.02	0.00	0.0056	0.0173	0.00
Thiamin	mg/kg	26.85	132.36	0.0356	8.21	8.93	0.002	139.36	154.85	0.04	45.32	140.50	0.04
Riboflavin	mg/kg	4.59	22.60	0.0061	10.49	11.40	0.003	17.98	19.97	0.01	5.81	18.02	0.01
Niacin	mg/kg	43.97	216.70	0.0583	73.50	79.89	0.022	204.92	227.69	0.06	66.37	205.74	0.06
Pantothenic acid	mg/kg	7.88	38.85	0.0105	41.76	45.39	0.013	23.86	26.51	0.01	7.71	23.91	0.01
Pyridoxine	mg/kg	5.87	28.94	0.0078	9.89	10.75	0.003	30.32	33.69	0.01	9.80	30.37	0.01
Folic acid	mg/kg	0.82	4.04	0.0011	1.00	1.08	0.000	3.52	3.91	0.00	1.15	3.55	0.00
Biotin	mg/kg	0.03	0.17	0.0000	0.22	0.24	0.000	0.18	0.19	0.00	0.0570	0.1768	0.00
Vitamin B12	mcg/kg	44.46	219.16	0.0001	71.08	77.26	0.000	82.47	91.64	0.00	26.68	82.71	0.00
Vitamin C	mg/kg	27.58	135.94	0.0366	N/A	N/A	N/A	187.59	208.43	0.06	60.35	187.09	0.06
Choline	mg/kg	760.14	3746.60	1.0079	1622.23	1763.30	0.486	3429.62	3810.70	1.02	1110.76	3443.34	1.02
Carnitine	mg/kg	206.30	1016.80	0.2735	N/A	N/A	N/A	1313.78	1459.76	0.39	422.72	1310.44	0.39
Alanine	%	0.51	2.51	6.74	1.45	1.58	4.35	2.67	2.97	7.93	0.86	2.67	7.92
Arginine	%	0.51	2.49	6.70	2.14	2.33	6.42	2.71	3.02	8.05	0.87	2.71	8.04
Aspartic acid	%	0.83	4.10	11.03	3.90	4.24	11.68	4.42	4.91	13.11	1.42	4.41	13.09
Glutamic acid	%	1.26	6.21	16.71	4.45	4.83	13.32	6.77	7.53	20.09	2.18	6.76	20.06
Glycine	%	0.49	2.39	6.43	1.42	1.54	4.25	2.58	2.87	7.66	0.83	2.58	7.65
Histidine	%	0.24	1.16	3.13	0.73	0.80	2.20	1.23	1.37	3.66	0.40	1.23	3.65
Isoleucine	%	0.39	1.91	5.13	1.51	1.64	4.51	2.01	2.23	5.95	0.65	2.00	5.94
Leucine	%	0.70	3.43	9.22	2.63	2.86	7.89	3.55	3.94	10.52	1.14	3.54	10.51
Lysine	%	0.71	3.48	9.36	2.36	2.56	7.06	3.80	4.22	11.26	1.22	3.79	11.25
Methionine	%	0.20	0.98	2.63	0.51	0.56	1.54	1.05	1.17	3.12	0.34	1.05	3.11
Met-Cystine	%	0.26	1.30	3.50	0.89	0.97	2.67	1.39	1.54	4.11	0.45	1.38	4.11
Phenylalanine	%	0.38	1.87	5.04	1.70	1.85	5.09	1.92	2.13	5.69	0.62	1.91	5.69
Phe-Tyrosine	%	0.68	3.34	8.98	2.92	3.18	8.76	3.45	3.84	10.24	1.11	3.44	10.23
Proline	%	0.38	1.88	5.05	1.41	1.53	4.22	2.00	2.22	5.92	0.64	1.99	5.92
Serine	%	0.37	1.83	4.93	1.52	1.65	4.54	1.93	2.14	5.71	0.62	1.92	5.71
Threonine	%	0.41	2.01	5.40	1.40	1.52	4.20	2.14	2.38	6.34	0.69	2.13	6.34
Tryptophan	%	0.11	0.55	1.49	0.36	0.39	1.08	0.57	0.63	1.69	0.18	0.57	1.69
Valine	%	0.41	2.04	5.50	1.73	1.88	5.19	2.11	2.35	6.26	0.68	2.11	6.26
Taurine	mg/kg	717.90	3538.41	0.95	526.95	572.77	0.16	2735.90	3039.89	0.81	886.34	2747.65	0.82
FOS	%	0.11	0.55	1.48	N/A	N/A	N/A	0.58	0.65	1.72	0.19	0.58	1.73

Abbreviations: AA, arachidonic acid; AAFCO, Association of American Feed Control Officials; AcDF, acid detergent fibre; ADF, moderately processed air-dried food; ALA, α-linoleic acid; DF, ultra-processed dry food; DHA, docosahexaenoic acid; EPA, eicosapentaenoic acid; FOS, fructooligosaccharide; LA, linoleic acid; LCPUFA, long-chain polyunsaturated fatty acid; ME, metabolizable energy; ME (AAFCO – 3.5, 8.5 kcal/kg) = (3.5 × crude protein) + (8.5 × crude fat); MF, minimally processed mildly cooked food; NDF, neutral detergent fibre.

**TABLE 4:** Method of processing and corresponding maximum temperatures employed during the manufacture of the four diets.

Diet	Type of processing	Methods	Maximum temperature °F (°C)	Comments
WF	Ultra-processed	Retorting	254 (123)	Exposed to maximum temperatures for 60–90 min
DF		Extrusion	265 (129)	After extrusion, food is dried to <10% moisture
ADF	Moderately processed	Dehydration	140 (60)	Dried for 12 h until <12% moisture
MF	Minimally processed	Cooked	105 (40)	Cooked for 10 h then frozen

Abbreviations: ADF, moderately processed air-dried food; DF, ultra-processed dry food; MF, minimally processed mildly cooked food; WF, ultra-processed wet food.

**TABLE 5:** Average amount (mg/100 kcal) as fed of individual and total dietary advanced glycation end products (AGEs) in the four differently processed diets.

AGEs	Diet			
	WF	DF	ADF	MF
CML	0.96	0.49	0.78	0.22
CEL	1.14	0.31	0.22	0.33
MG-H1	0.54	0.40	0.36	0.77
CML + CEL	2.10	0.80	1.00	0.55
CML + CEL + MG-H1	2.64	1.20	1.36	1.31

Abbreviations: ADF, moderately processed air-dried food; CEL, N^ε^-(carboxyethyl)lysine; CML, N^ε^-(carboxymethyl)lysine; DF, ultra-processed dry food; MF, minimally processed mildly cooked food; MG-H1, methylglyoxal hydroimidazolone-1; WF, ultra-processed wet food.

**TABLE 6a:** Average plasma concentration (nM/50 μL) of individual advanced glycation end products (AGEs) at the end of feeding (4 weeks) the four differently processed diets: Canned wet, dry kibble, air-dried and mildly cooked.

AGE	Diet			
	WF	DF	ADF	MF
CML	1.50 (1.24–1.85)^a^	1.78 (1.12–2.51)^a^	2.34 (1.54–3.91)^a^	0.77 (0.61–0.91)^b^
CEL	2.45 (1.67–3.23)^a^	1.54 (0.85–1.72)^b^	0.64 (0.51–1.03)^b^	0.77 (0.61–1.07)^b^
GH-1	0.08 (0.07–0.09)^a^	0.07 (0.06–0.07)^ab^	0.09 (0.07–0.12)^a^	0.05 (0.04–0.05)^b^
MG-H1	5.41 (3.39–7.40)^a^	1.53 (0.61–3.26)^b^	1.01 (0.61–2.12)^bc^	0.37 (0.18–0.57)^bd^
AP	0.46 (0.32–0.56)^a^	0.35 (0.32–0.45)^a^	0.34 (0.29–0.38)^a^	0.62 (0.52–0.69)^b^

*Note*: Values are medians with interquartile ranges. Statistically significant differences indicated by superscript letters—a, b, c and d as determined by Friedman’s tests and Wilcoxon Mann–Whitney post-hoc individual comparisons (*p* < 0.05).

Abbreviations: ADF, moderately processed air-dried food; AP, argpyrimidine; CEL, N^ε^-(carboxyethyl)lysine; CML, N^ε^-(carboxymethyl)lysine; DF, ultra-processed dry food; GH-1, glyoxal hydroimidazolone-1; MF, minimally processed mildly cooked food; MG-H1, methylglyoxal hydroimidazolone-1; WF, ultra-processed wet food.

**TABLE 6b:** Average plasma concentration (nM/50 μL) of grouped and total advanced glycation end products (AGEs) at the end of feeding (4 weeks) the four differently processed diets.

AGEs grouped	Diet			
	WF	DF	ADF	MF
CML + CEL	4.04 (2.94–5.04)^a^	3.44 (1.98–4.09)^a^	3.12 (2.05–4.82)^a^	1.46 (1.36–1.92)^b^
GH-1+ MG-H1	5.49 (3.49–7.46)^a^	1.59 (0.68–3.32)^b^	1.13 (0.68–2.24)^bc^	0.42 (0.22–0.64)^bd^
CML + CEL + GH-1+ MG-H1	9.57 (6.43–12.51)^a^	5.67 (2.65–6.81)^b^	4.21 (2.77–7.00)^bc^	1.89 (1.64–2.48)^bd^
Total (CML + CEL + GH-1+ MG-H1 + AP)	9.99 (6.74–12.95)^a^	6.01 (3.10–7.14)^b^	4.53 (3.12–7.42)^bc^	2.50 (2.33–3.14)^bd^

*Note*: Values are medians with interquartile ranges. Statistically significant differences indicated by superscript letters—a, b, c and d as determined by Friedman’s tests and Wilcoxon Mann–Whitney post hoc multiple comparisons (*p* < 0.05).

Abbreviations: ADF, moderately processed air-dried food; AP, argpyrimidine; CEL, Nε-(carboxyethyl)lysine; CML, Nε-(carboxymethyl)lysine; DF, ultra-processed dry food; GH-1, glyoxal hydroimidazolone-1; MF, minimally processed mildly cooked food; MG-H1, methylglyoxal hydroimidazolone-1; WF, ultra-processed wet food.

**TABLE 7a:** Significant differences between diets for individual plasma advanced glycation end products (AGEs).

AGE	Diet contrast	Hodges–Lehmann location shift	95% Confidence interval (CI)	*p* Value
CML	ADF – MF	1.30	0.65–3.62	0.0046
	ADF – WF	0.75	−0.20 to 2.73	0.1152
	ADF – DF	0.65	−0.69 to 2.56	0.3446
	DF – MF	0.97	0.09–1.77	0.0357
	WF – MF	0.68	0.40–1.09	0.0046
	DF – WF	0.29	−0.48 to 1.15	0.4008
CEL	WF – ADF	1.55	0.86–2.62	0.0016
	WF – MF	1.41	0.77–2.52	0.0016
	WF – DF	1.21	0.02–1.85	0.0460
	DF – ADF	0.64	−0.05 to 1.21	0.0742
	DF – MF	0.61	−0.13 to 1.06	0.0929
	MF – ADF	0.11	−0.29 to 0.51	0.5995
GH-1	ADF – MF	0.04	0.20–0.07	0.0033
	WF – MF	0.03	0.01–0.05	0.0274
	ADF – DF	0.03	−0.01 to 0.06	0.1415
	DF – MF	0.02	−0.01 to 0.03	0.0587
	ADF – WF	0.02	−0.01 to 0.05	0.3446
	WF – DF	0.01	−0.01 to 0.03	0.1152
MG-H1	WF – MF	4.96	2.18–7.19	0.0016
	WF – ADF	4.26	1.77–6.57	0.0033
	WF – DF	3.84	1.44–6.38	0.0063
	DF – MF	0.97	−0.03 to 2.92	0.0587
	ADF – MF	0.64	0.01–2.01	0.0460
	DF – ADF	0.46	−0.79 to 2.25	0.4622
AP	MF – ADF	0.28	0.17–0.38	0.0011
	MF – DF	0.25	0.12–0.35	0.0033
	MF – WF	0.17	0.04–0.33	0.0117
	WF – ADF	0.09	−0.03 to 0.23	0.1722
	WF – DF	0.03	−0.06 to 0.22	0.2076
	DF – ADF	0.02	−0.04 to 0.14	0.4622

*Note*: Statistically significant differences determined by Friedman’s tests and Wilcoxon Mann Whitney post hoc individual comparisons (*p* < 0.05). Abbreviations: ADF, moderately processed air-dried food; AP, argpyrimidine; CEL, N^ε^-(carboxyethyl)lysine; CML, N^ε^-(carboxymethyl)lysine; DF, ultra-processed dry food; GH-1, glyoxal hydroimidazolone-1; MF, minimally processed mildly cooked food; MG-H1, methylglyoxal hydroimidazolone-1; WF, ultra-processed wet food.

**TABLE 7b:** Significant differences between diets for grouped and total plasma advanced glycation end products (AGEs).

AGE	Diet contrast	Hodges–Lehmann location shift	95% Confidence interval (CI)	*p* Value
CML + CEL	WF – MF	1.87	1.33–3.58	0.0033
	DF – MF	1.73	0.12–2.68	0.0460
	ADF – MF	1.18	0.24–3.97	0.0357
	WF – DF	0.95	−0.96 to 2.37	0.4008
	WF – ADF	0.88	−0.96 to 2.52	0.4008
	ADF – DF	0.01	−1.81 to 2.13	1.0000
GH-1+ MG-H1	WF – MF	4.97	2.22–7.22	0.0016
	WF – ADF	4.25	1.76–6.53	0.0033
	WF – DF	3.82	1.35–6.39	0.0063
	DF – MF	0.99	−0.05 to 2.95	0.0742
	ADF – MF	0.68	0.04–2.03	0.0460
	DF – ADF	0.43	−0.87 to 2.18	0.4622
CML + CEL + GH-1+ MG-H1	WF – MF	6.74	3.56–10.51	0.0016
	WF – ADF	4.70	1.08–9.14	0.0157
	WF – DF	4.68	0.70–8.68	0.0274
	DF – MF	3.53	−0.06 to 4.98	0.0587
	ADF – MF	1.92	0.38–5.15	0.0357
	DF – ADF	0.14	−3.13 to 3.52	1.0000
CML + CEL + GH-1+ MG-H1 + AP	WF – MF	6.59	3.28–10.55	0.0016
	WF – ADF	4.89	0.97–9.16	0.0157
	WF – DF	4.53	0.71–8.53	0.0460
	DF – MF	3.24	−0.24 to 4.72	0.0929
	ADF – MF	1.70	0.02–4.95	0.0460
	DF – ADF	0.09	−3.30 to 3.49	1.0000

*Note*: Statistically significant differences determined by Friedman’s tests and Wilcoxon Mann–Whitney post hoc individual comparisons (*p* < 0.05). Abbreviations: ADF, moderately processed air-dried food; AP, argpyrimidine; CEL, N^ε^-(carboxyethyl)lysine; CML, N^ε^-(carboxymethyl)lysine; DF, ultra-processed dry food; GH-1, glyoxal hydroimidazolone-1; MF, minimally processed mildly cooked food; MG-H1, methylglyoxal hydroimidazolone-1; WF, ultra-processed wet food.

**TABLE 8:** Table showing advanced glycation end products measured in urine expressed as AGE nmol/mmol urine creatinine.

AGE	Diet			
	WF	DF	ADF	MF
CML	5547.59^a^ (4183.37–10,189.29)	13,703.65^b^ (12,507.88–21,961.45)	8829.92^ab^ (7130.88–33,028.77)	3282.69^a^ (2447.52–13,329.59)
CEL	9387.81 (9047.74–15,852.44)	8010.04 (7471.27–14,398.95)	2630.52 (1676.87–8613.16)	5495.05 (3398.76–10,574.92)
LAL	3132.54 (1365.84–8818.18)	1387.66 (1149.47–3208.75)	1058.52 (640.79–34,124.87)	2453.13 (1546.46–14,537.42)
CML + CEL	15,053.60 (14,364.47–27,261.28	21,527.42 (20,072.28–36,524.13)	11,460.43 (8900.92–41,641.92)	7706.49 (5846.29–23,691.12)
CML + CEL + LAL	21,697.84 (15,662.64–34,235.73)	23,507.26 (21,919.28–37,820.53)	16,965.22 (9560.72–72,927.23)	16,084.94 (8933.13–29,435.55)

*Note*: Values are displayed as medians and interquartile ranges. Statistically significant differences indicated by superscript letters – a and b as determined by Friedman’s tests and Wilcoxon Signed Rank post-hoc individual comparisons (*p* < 0.05).

Abbreviations: ADF, moderately processed air-dried food; CEL, N^ε^-(carboxyethyl)lysine; CML, N^ε^-(carboxymethyl)lysine; DF, ultra-processed dry food; LAL, lysinoalanine; MF, minimally processed mildly cooked food; WF, ultra-processed wet food.

**TABLE 9:** Table showing statistically significant differences in urine concentration of advanced glycation end products in urine.

AGE	Diets	*Z* value	*p* Value
CML	DF – WF	−2.240	0.025
	ADF – WF	−1.960	0.050
	MF – WF	−0.338	0.735
	ADF – DF	0.000	1.000
	MF – DF	−2.197	0.028
	MF – ADF	−1.859	0.063
CEL	DF – WF	0.000	1.000
	ADF – WF	−1.680	0.093
	MF – WF	−0.338	0.735
	ADF – DF	−1.400	0.161
	MF – DF	−0.676	0.499
	MF – ADF	−1.690	0.091
LAL	DF – WF	−0.980	0.327
	ADF – WF	−0.280	0.779
	MF – WF	−0.338	0.735
	ADF – DF	−0.560	0.575
	MF – DF	−1.521	0.128
	MF – ADF	−0.338	0.735
CML + CEL	DF – WF	−1.400	0.161
	ADF – WF	−0.140	0.889
	MF – WF	−0.338	0.735
	ADF – DF	−0.280	0.779
	MF – DF	−1.183	0.237
	MF – ADF	0.000	1.000
CML + CEL + LAL	DF – WF	−0.700	0.484
	ADF – WF	−0.140	0.889
	MF – WF	−0.676	0.499
	ADF – DF	−0.420	0.674
	MF – DF	−1.014	0.310
	MF – ADF	−0.169	0.866

*Note*: Statistically significant differences determined by Friedman’s tests and Wilcoxon signed-rank post hoc individual comparisons (*p* < 0.05).

Abbreviations: ADF, moderately processed air-dried food; CEL, N^ε^-(carboxyethyl)lysine; CML, N^ε^-(carboxymethyl)lysine; DF, ultra-processed dry food; LAL, lysinoalanine; MF, minimally processed mildly cooked food; WF, ultra-processed wet food.

**TABLE 10a:** Soluble receptor for advanced glycation end products (sRAGE) concentration (pg/mL) medians, interquartile ranges and mean rank values for serum at the end of feeding each diet.

Diet	Median	Interquartile range	Mean rank (Friedman’s test)
WF	603.42	280.38–3006.02	2.50
DF	460.11	198.42–2908.24	1.63
ADF	651.42	286.09–2829.24	2.50
MF	690.35	317.01–3881.52	3.38

Abbreviations: ADF, moderately processed air-dried food; DF, ultra-processed dry food; MF, minimally processed mildly cooked food; WF, ultra-processed wet food.

**TABLE 10b:** Total advanced glycation end products to soluble receptor for advanced glycation end products (AGE/sRAGE) ratio based on plasma total AGE concentration (nM/mL) against sRAGE concentration (pg/mL).

Diet comparisons	*Z* value	*p* value
DF – WF	−1.120	0.263
ADF – WF	−1.540	0.123
MF – WF	−2.366	0.018
ADF – DF	−2.100	0.036
MF – DF	−1.859	0.063
MF – ADF	−1.690	0.091

*Note*: Statistically significant differences determined by Friedman’s tests and Wilcoxon signed-rank post hoc individual comparisons (*p* < 0.05).

All *Z* values based on positive ranks.

## Data Availability

The data that support the findings of this study are available from the corresponding author upon reasonable request.
