# Supplementary material for: Association of four differently processed diets with plasma and urine advanced glycation end products and serum soluble receptor for advanced glycation end products concentration in healthy dogs
Source: J Anim Physiol Anim Nutr (Berl). Author manuscript; Available in PMC 2024 Aug 16. (PMC11327896; doi:10.1111/jpn.13927)
Supplement: Supp for healthy dogs [file NIHMS2009800-supplement-Supp_for_healthy_dogs.pdf]

Table S1. Clinical parameters calculated as significantly different between diets: ultra-processed wet food (WF), ultra-processed dry food (DF), moderately processed air-dried food (ADF), and minimally processed, mildly cooked food (MF).

**Parameter**	**WF**	**DF**	**ADF**	**MF**
Aspartate transaminase (U/L)	24.50^{a} (19.75 – 27.00)	22.00^{ab} (16.75 – 24.75	22.50^{ab} (20.00 – 28.00)	25.50^{ac} (23.50 – 31.50)
Alanine transferase (U/L)	32.00^{a} (28.00 – 47.75)	30.50^{ab} (26.00 – 40.25)	34.50^{a} (29.50 – 38.25	44.00^{ac} (32.00 – 72.75)
Alkaline phosphatase (U/L)	41.50^{a} (25.25 – 91.00	78.00^{ab} (43.00 – 107.00)	41.00^{ac} (26.25 – 54.00)	66.00^{ab} (60.25 – 78.50
Creatinine (mg/dL)	0.60^{a} (0.60 – 0.75)	0.50^{b} (0.43 – 0.58)	0.60^{a} (0.60 – 0.70)	0.60^{a} (0.53 – 0.70
Phosphorus (mg/dL)	3.30^{a} (2.98 – 3.58)	4.55^{b} (4.13 – 5.40)	3.65^{a} (3.13 – 4.58)	3.45^{a} (3.00 – 3.90)
Chloride (mEq/L)	113.50^{a} (113.00 – 114.00)	112.00^{a} (111.00 – 114.00)	114.00^{b} (113.25 – 116.50)	113.00^{a} (109.75 – 113.75)
Cholesterol (mg/dL)	165.00^{a} (158.00 – 198.25)	168.50^{a} (139.00 – 215.00)	175.00^{a} (150.75 – 198.50)	213.50^{b} (184.25 – 261.750
Hemoglobin (g/dL)	15.05^{a} (14.53 – 17.13)	14.00^{b} (13.63 – 15.50)	15.25^{ab} (14.10 – 17.380	16.25^{a} (14.20 – 17.35)
Urine pH	6.25^{a} (6.00 – 7.25)	8.25^{b} (8.00 – 8.50)	8.5^{bc} (7.38 – 8.50)	7.50^{ac} (7.00 – 8.38)
Urine specific gravity	1.024^{a} (1.022 – 1.028)	1.041^{b} (1.037 – 1.053)	1.050^{b} (1.043 – 1.055)	1.023^{ac} (1.022 – 1.025)

† Values shown are median (interquartile range)

‡ Statistically significant differences are indicated by superscript letters a, b and c as determined by Friedman's tests and Wilcoxon Signed Rank post-hoc individual comparisons ($p < 0.05$).
